# 3-Ethyl-4-hy­droxy-8-meth­oxy­quinolin-2(1*H*)-one

**DOI:** 10.1107/S1600536812043279

**Published:** 2012-10-24

**Authors:** Stanislav Kafka, Andrej Pevec, Karel Proisl, Roman Kimmel, Janez Košmrlj

**Affiliations:** a Department of Chemistry, Faculty of Technology, Tomas Bata University in Zlin, Zlin 76272, Czech Republic; b Faculty of Chemistry and Chemical Technology, University of Ljubljana, SI-1000 Ljubljana, Slovenia

## Abstract

In the title compound, C_12_H_13_NO_3_, the quinoline ring system is approximately planar with a maximum deviation from the least-squares plane of 0.058 (2) Å. In the crystal, N—H⋯O and O—H⋯O hydrogen bonds link the mol­ecules into chains running along the *b*-axis direction. The chains also feature π–π inter­actions between pyridine and benzene rings of inversion-related mol­ecules [centroid–centroid distance = 3.609 (2) Å].

## Related literature

For naturally occurring 3-alkyl-4-hy­droxy­quinolin-2-ones, see: Paul & Bose (1968 ▶); Faizutdinova *et al.* (1969 ▶); Jurd *et al.* (1983 ▶); Chen *et al.* (1994 ▶); Yamamoto & Harimaya (2004 ▶); Jain *et al.* (2006 ▶). For the first published synthesis of the title compound, see: Rapoport & Holden (1959 ▶). For recent synthetic utilization of 3-alkyl-4-hy­droxy­quinolin-2-ones, see, for example: Kimmel *et al.* (2010 ▶).
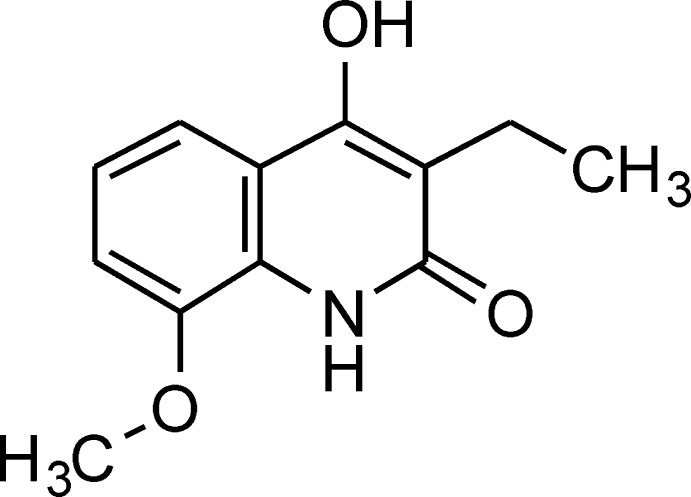


## Experimental

### 

#### Crystal data

C_12_H_13_NO_3_
*M* *_r_* = 219.23
Monoclinic, 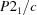

*a* = 11.4824 (4) Å
*b* = 6.9072 (2) Å
*c* = 14.4978 (5) Å
β = 113.1283 (15)°
*V* = 1057.42 (6) Å^3^
*Z* = 4
Mo *K*α radiation
μ = 0.10 mm^−1^
*T* = 293 K
0.35 × 0.25 × 0.08 mm

#### Data collection

Nonius KappaCCD area-detector diffractometer
Absorption correction: multi-scan (*SCALEPACK*; Otwinowski & Minor, 1997 ▶) *T*_min_ = 0.966, *T*_max_ = 0.992
4558 measured reflections
2403 independent reflections
1734 reflections with *I* > 2σ(*I*)
*R*_int_ = 0.025

#### Refinement

*R*[*F* ^2^ > 2σ(*F* ^2^)] = 0.077
*wR*(*F* ^2^) = 0.272
*S* = 1.14
2403 reflections
151 parameters
1 restraint
H atoms treated by a mixture of independent and constrained refinement
Δρ_max_ = 0.37 e Å^−3^
Δρ_min_ = −0.29 e Å^−3^

### 

Data collection: *COLLECT* (Nonius, 1998 ▶); cell refinement: *DENZO* and *SCALEPACK* (Otwinowski & Minor, 1997 ▶); data reduction: *DENZO* and *SCALEPACK*; program(s) used to solve structure: *SHELXS97* (Sheldrick, 2008 ▶); program(s) used to refine structure: *SHELXL97* (Sheldrick, 2008 ▶); molecular graphics: *PLATON* (Spek, 2009 ▶) and *DIAMOND* (Brandenburg, 1999 ▶); software used to prepare material for publication: *WinGX* (Farrugia, 1999 ▶).

## Supplementary Material

Crystal structure: contains datablock(s) I, global. DOI: 10.1107/S1600536812043279/tk5161sup1.cif

Structure factors: contains datablock(s) I. DOI: 10.1107/S1600536812043279/tk5161Isup2.hkl

Supplementary material file. DOI: 10.1107/S1600536812043279/tk5161Isup3.cml

Additional supplementary materials:  crystallographic information; 3D view; checkCIF report

## Figures and Tables

**Table 1:** Hydrogen-bond geometry (Å, °)

*D*—H⋯*A*	*D*—H	H⋯*A*	*D*⋯*A*	*D*—H⋯*A*
N1—H1*N*⋯O1^i^	0.85 (2)	2.27 (3)	2.976 (4)	140 (3)
O2—H2⋯O1^ii^	0.82	1.94	2.665 (4)	147

Symmetry codes: (i) 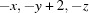; (ii) 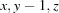.

**Fig. 1.**
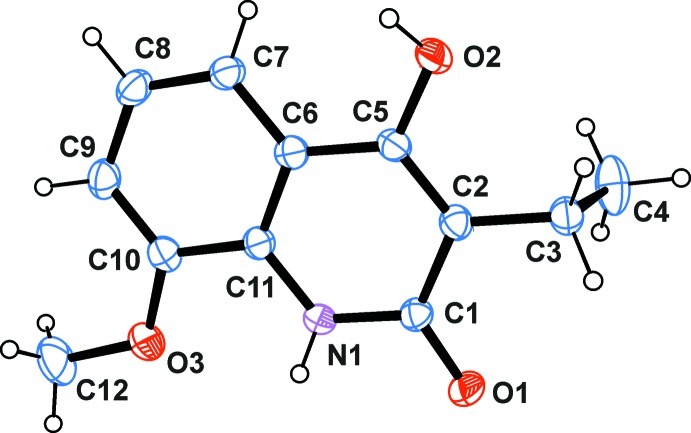
A view of the title compound showing the atom-numbering scheme. Displacement ellipsoids are drawn at the 30% probability level.

**Fig. 2.**
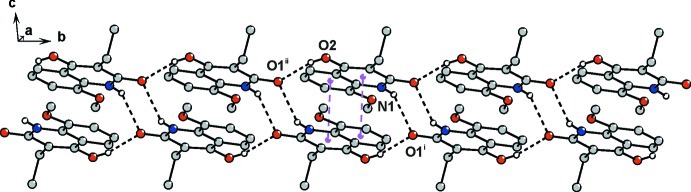
The supramolecular chain in the crystal structure of (I), with the O—H···O and N—H···O hydrogen bonds, and π–π interactions denoted by dashed lines. Hydrogen atoms not involved in hydrogen bonding have been omitted for clarity. [Symmetry codes: (i) -x, -y + 2, -z; (ii) x, y - 1, z]

## supplementary crystallographic information

### Comment

The title compound, (I) (Fig. 1), was recently prepared as an intermediate within the framework of a study focusing on glucosylation of *N*-unsubstituted 4-hydroxyquinolin-2(1*H*)-ones by thermal condensation of diethyl ethylmalonate with *o*-anisidine (Kimmel *et al.*, 2010). Some 3-alkyl-4-hydroxyquinolin-2-ones were isolated from plants *Ravenia spectabilis* (Paul & Bose, 1968), *Haplophylum bucharicum* (Faizutdinova *et al.*, 1969), *Euxylophora pareansis* (Jurd *et al.*, 1983), *Zanthoxylum simulans* (Chen *et al.*, 1994), *Toddalia aculeata* (Jain *et al.*, 2006) as well as from the fermentation broth of *Dactylosporangium sp*. (Yamamoto & Harimaya, 2004).

In the crystal structure of the title compound (I) (Fig. 2) two 3-ethyl-4-hydroxy-8-methoxyquinolin-2(1*H*)-one molecules are connected by two intermolecular N—H···O hydrogen bonds between protonated nitrogen atom and carbonyl group. These connections altogether with additional O—H···O hydrogen bonds between hydroxyl and carbonyl groups (Table 1) form linear chain along *b* axis. The chains are further stabilized by *π*–*π* interactions between pyridine and benzene rings of inversion-related pairs of quinoline molecules [centroid–centroid distance = 3.609 (2) Å].

### Experimental

A mixture of *o*-anisidine (12.3 g, 100 mmol) and diethyl ethylmalonate (197.6 g, 105 mmol) was heated on a metal bath at 220–230 °C for 1 h and then at 260–270 °C for 6 h (until the distillation of ethanol stopped). The hot reaction mixture was cautiously poured into toluene (50 ml). After cooling, the precipitate was filtered. The residue was dissolved in aqueous sodium hydroxide solution (0.5 *M*, 300 ml) and the solution was filtered. The filtrate was washed with toluene (3 *x* 15 ml). The aqueous phase was filtered and acidified by addition of 10% hydrochloric acid to Congo red. The precipitated paste was triturated with a glass bar under an aqueous phase for several minutes and then the mixture was cooled in refrigerator for several hours, until the pasty substance hardened. The solid was filtered off, washed with water (100 ml), air dried and crystallized from ethanol affording 13.6 g (62% of theoretical yield) of the title compound (I), m. pt 496–498 K (benzene – ethanol). In the literature (Rapoport & Holden, 1959), a m. pt range of 498–499 K is reported.

### Refinement

The N–bonded hydrogen atom was located in a difference map and refined with the using a distance restraint, N—H = 0.86±0.02 Å, and with *U*_iso_(H) = 1.2*U*_eq_(N). All other H atoms were included in the model at geometrically calculated positions and refined using a riding model, with C—H bond lengths constrained to 0.93 Å (aromatic H), 0.96 Å (methyl H), 0.97 Å (methylene H) and O—H = 0.82 Å, and with *U*_iso_(H) values of 1.2*U*_eq_(C) [for aromatic and methylene H] or 1.5*U*_eq_(C) [for oxygen and methyl H]. The exceptionally large value for the first parameter on the *SHELXL* weighting line altogether with large value for weighted *R* factor indicate possible twinning. The function TwinRotMat in *PLATON* (Spek, 2009) suggests that the structure could be twinned. However, applying the proposed twin law does not affect the refinement in the sense of better *R* values. Additionally, the BASF parameter has a value close to zero after refinement. Hence, a twin model was not employed.

### Crystal data

**Table:**

C_12_H_13_NO_3_	*F*(000) = 464
*M**_r_* = 219.23	*D*_x_ = 1.377 Mg m^−^^3^
Monoclinic, *P*2_1_/*c*	Melting point = 496–498 K
Hall symbol: -P 2ybc	Mo *K*α radiation, λ = 0.71073 Å
*a* = 11.4824 (4) Å	Cell parameters from 2554 reflections
*b* = 6.9072 (2) Å	θ = 0.4–27.5°
*c* = 14.4978 (5) Å	µ = 0.10 mm^−^^1^
β = 113.1283 (15)°	*T* = 293 K
*V* = 1057.42 (6) Å^3^	Prism, colourless
*Z* = 4	0.35 × 0.25 × 0.08 mm

### Data collection

**Table:**

Nonius KappaCCD area-detector diffractometer	2403 independent reflections
Radiation source: fine-focus sealed tube	1734 reflections with *I* > 2σ(*I*)
Graphite monochromator	*R*_int_ = 0.025
φ scans + ω scans	θ_max_ = 27.5°, θ_min_ = 3.1°
Absorption correction: multi-scan (*SCALEPACK*; Otwinowski & Minor, 1997)	*h* = −14→14
*T*_min_ = 0.966, *T*_max_ = 0.992	*k* = −8→8
4558 measured reflections	*l* = −18→18

### Refinement

**Table:**

Refinement on *F*^2^	Primary atom site location: structure-invariant direct methods
Least-squares matrix: full	Secondary atom site location: difference Fourier map
*R*[*F*^2^ > 2σ(*F*^2^)] = 0.077	Hydrogen site location: inferred from neighbouring sites
*wR*(*F*^2^) = 0.272	H atoms treated by a mixture of independent and constrained refinement
*S* = 1.14	*w* = 1/[σ^2^(*F*_o_^2^) + (0.1076*P*)^2^ + 1.7454*P*] where *P* = (*F*_o_^2^ + 2*F*_c_^2^)/3
2403 reflections	(Δ/σ)_max_ = 0.0001
151 parameters	Δρ_max_ = 0.37 e Å^−^^3^
1 restraint	Δρ_min_ = −0.29 e Å^−^^3^

### Special details

**Table:**

Experimental. 279 frames in 4 sets of φ scans + ω scans. Rotation/frame = 1.6 °. Crystal-detector distance = 32 mm. Measuring time = 150 s/°.
Geometry. All s.u.'s (except the s.u. in the dihedral angle between two l.s. planes) are estimated using the full covariance matrix. The cell s.u.'s are taken into account individually in the estimation of s.u.'s in distances, angles and torsion angles; correlations between s.u.'s in cell parameters are only used when they are defined by crystal symmetry. An approximate (isotropic) treatment of cell s.u.'s is used for estimating s.u.'s involving l.s. planes.
Refinement. Refinement of *F*^2^ against ALL reflections. The weighted *R*-factor *wR* and goodness of fit *S* are based on *F*^2^, conventional *R*-factors *R* are based on *F*, with *F* set to zero for negative *F*^2^. The threshold expression of *F*^2^ > 2σ(*F*^2^) is used only for calculating *R*-factors(gt) *etc*. and is not relevant to the choice of reflections for refinement. *R*-factors based on *F*^2^ are statistically about twice as large as those based on *F*, and *R*- factors based on ALL data will be even larger.

### Fractional atomic coordinates and isotropic or equivalent isotropic displacement parameters (Å^2^)

**Table:**

	*x*	*y*	*z*	*U*_iso_*/*U*_eq_	
N1	0.0025 (3)	0.7822 (4)	0.0870 (2)	0.0373 (7)	
H1N	−0.045 (3)	0.880 (4)	0.064 (3)	0.045*	
O1	0.1611 (2)	0.9727 (3)	0.0836 (2)	0.0482 (7)	
O2	0.2406 (2)	0.3155 (4)	0.1696 (2)	0.0506 (7)	
H2	0.1991	0.2151	0.1570	0.076*	
O3	−0.2344 (2)	0.7758 (4)	0.0700 (2)	0.0584 (8)	
C1	0.1260 (3)	0.8088 (5)	0.1009 (3)	0.0362 (7)	
C2	0.2089 (3)	0.6432 (5)	0.1318 (3)	0.0370 (8)	
C3	0.3448 (3)	0.6709 (6)	0.1462 (3)	0.0437 (9)	
H3A	0.3497	0.7733	0.1020	0.052*	
H3B	0.3757	0.5528	0.1275	0.052*	
C4	0.4286 (4)	0.7222 (9)	0.2532 (4)	0.0740 (15)	
H4A	0.4028	0.8447	0.2702	0.111*	
H4B	0.5150	0.7303	0.2599	0.111*	
H4C	0.4212	0.6241	0.2975	0.111*	
C5	0.1612 (3)	0.4696 (5)	0.1440 (2)	0.0359 (7)	
C6	0.0321 (3)	0.4481 (5)	0.1348 (2)	0.0356 (7)	
C7	−0.0181 (3)	0.2749 (5)	0.1554 (3)	0.0407 (8)	
H7	0.0325	0.1651	0.1758	0.049*	
C8	−0.1418 (4)	0.2694 (6)	0.1450 (3)	0.0473 (9)	
H8	−0.1749	0.1547	0.1580	0.057*	
C9	−0.2195 (3)	0.4337 (6)	0.1151 (3)	0.0469 (9)	
H9	−0.3035	0.4269	0.1080	0.056*	
C10	−0.1721 (3)	0.6033 (6)	0.0964 (3)	0.0412 (8)	
C11	−0.0451 (3)	0.6117 (5)	0.1052 (2)	0.0353 (7)	
C12	−0.3645 (4)	0.7815 (8)	0.0518 (4)	0.0780 (16)	
H12A	−0.4091	0.6864	0.0022	0.117*	
H12B	−0.3977	0.9078	0.0281	0.117*	
H12C	−0.3753	0.7540	0.1129	0.117*	

### Atomic displacement parameters (Å^2^)

**Table:**

	*U*^11^	*U*^22^	*U*^33^	*U*^12^	*U*^13^	*U*^23^
N1	0.0334 (14)	0.0319 (14)	0.0455 (16)	0.0049 (11)	0.0145 (12)	0.0054 (12)
O1	0.0463 (14)	0.0305 (13)	0.0695 (18)	−0.0029 (10)	0.0246 (13)	0.0036 (12)
O2	0.0354 (13)	0.0334 (13)	0.0717 (18)	0.0040 (10)	0.0091 (12)	−0.0001 (13)
O3	0.0389 (14)	0.0581 (18)	0.081 (2)	0.0121 (12)	0.0263 (14)	0.0145 (15)
C1	0.0368 (16)	0.0330 (17)	0.0390 (17)	−0.0012 (13)	0.0150 (14)	−0.0018 (13)
C2	0.0339 (16)	0.0361 (17)	0.0394 (17)	−0.0012 (13)	0.0127 (13)	−0.0042 (14)
C3	0.0351 (17)	0.0407 (19)	0.055 (2)	−0.0013 (14)	0.0170 (15)	−0.0026 (16)
C4	0.040 (2)	0.103 (4)	0.073 (3)	−0.020 (2)	0.016 (2)	−0.031 (3)
C5	0.0339 (16)	0.0325 (16)	0.0357 (16)	0.0027 (13)	0.0078 (13)	−0.0011 (13)
C6	0.0377 (16)	0.0358 (17)	0.0291 (15)	−0.0024 (13)	0.0084 (13)	−0.0026 (13)
C7	0.0445 (19)	0.0371 (18)	0.0388 (18)	−0.0023 (14)	0.0144 (14)	0.0037 (15)
C8	0.050 (2)	0.046 (2)	0.048 (2)	−0.0106 (17)	0.0208 (17)	0.0061 (17)
C9	0.0376 (18)	0.059 (2)	0.047 (2)	−0.0065 (16)	0.0197 (15)	0.0021 (18)
C10	0.0362 (17)	0.048 (2)	0.0388 (17)	0.0028 (15)	0.0143 (14)	0.0013 (15)
C11	0.0349 (16)	0.0359 (17)	0.0330 (16)	−0.0012 (13)	0.0110 (13)	−0.0006 (13)
C12	0.039 (2)	0.083 (4)	0.108 (4)	0.017 (2)	0.025 (2)	0.000 (3)

### Geometric parameters (Å, º)

**Table:**

N1—C1	1.364 (4)	C4—H4B	0.9600
N1—C11	1.367 (4)	C4—H4C	0.9600
N1—H1N	0.852 (19)	C5—C6	1.443 (4)
O1—C1	1.260 (4)	C6—C11	1.396 (5)
O2—C5	1.355 (4)	C6—C7	1.409 (5)
O2—H2	0.8200	C7—C8	1.369 (5)
O3—C10	1.365 (5)	C7—H7	0.9300
O3—C12	1.412 (5)	C8—C9	1.403 (6)
C1—C2	1.442 (5)	C8—H8	0.9300
C2—C5	1.359 (5)	C9—C10	1.363 (5)
C2—C3	1.503 (4)	C9—H9	0.9300
C3—C4	1.512 (6)	C10—C11	1.415 (5)
C3—H3A	0.9700	C12—H12A	0.9600
C3—H3B	0.9700	C12—H12B	0.9600
C4—H4A	0.9600	C12—H12C	0.9600
			
C1—N1—C11	123.9 (3)	C2—C5—C6	122.1 (3)
C1—N1—H1N	115 (3)	C11—C6—C7	119.3 (3)
C11—N1—H1N	121 (3)	C11—C6—C5	116.7 (3)
C5—O2—H2	109.5	C7—C6—C5	123.9 (3)
C10—O3—C12	118.4 (3)	C8—C7—C6	119.5 (3)
O1—C1—N1	119.1 (3)	C8—C7—H7	120.2
O1—C1—C2	123.4 (3)	C6—C7—H7	120.2
N1—C1—C2	117.4 (3)	C7—C8—C9	121.2 (3)
C5—C2—C1	119.3 (3)	C7—C8—H8	119.4
C5—C2—C3	123.0 (3)	C9—C8—H8	119.4
C1—C2—C3	117.7 (3)	C10—C9—C8	120.1 (3)
C2—C3—C4	112.3 (3)	C10—C9—H9	120.0
C2—C3—H3A	109.1	C8—C9—H9	120.0
C4—C3—H3A	109.1	C9—C10—O3	126.9 (3)
C2—C3—H3B	109.1	C9—C10—C11	119.7 (3)
C4—C3—H3B	109.1	O3—C10—C11	113.4 (3)
H3A—C3—H3B	107.9	N1—C11—C6	120.2 (3)
C3—C4—H4A	109.5	N1—C11—C10	119.6 (3)
C3—C4—H4B	109.5	C6—C11—C10	120.1 (3)
H4A—C4—H4B	109.5	O3—C12—H12A	109.5
C3—C4—H4C	109.5	O3—C12—H12B	109.5
H4A—C4—H4C	109.5	H12A—C12—H12B	109.5
H4B—C4—H4C	109.5	O3—C12—H12C	109.5
O2—C5—C2	117.8 (3)	H12A—C12—H12C	109.5
O2—C5—C6	120.0 (3)	H12B—C12—H12C	109.5
			
C11—N1—C1—O1	−178.8 (3)	C5—C6—C7—C8	179.6 (3)
C11—N1—C1—C2	3.3 (5)	C6—C7—C8—C9	−0.6 (5)
O1—C1—C2—C5	−176.7 (3)	C7—C8—C9—C10	−0.4 (6)
N1—C1—C2—C5	1.0 (5)	C8—C9—C10—O3	−177.8 (4)
O1—C1—C2—C3	1.6 (5)	C8—C9—C10—C11	1.4 (5)
N1—C1—C2—C3	179.4 (3)	C12—O3—C10—C9	−5.3 (6)
C5—C2—C3—C4	−89.5 (5)	C12—O3—C10—C11	175.5 (4)
C1—C2—C3—C4	92.3 (4)	C1—N1—C11—C6	−3.5 (5)
C1—C2—C5—O2	177.8 (3)	C1—N1—C11—C10	174.7 (3)
C3—C2—C5—O2	−0.5 (5)	C7—C6—C11—N1	178.6 (3)
C1—C2—C5—C6	−5.1 (5)	C5—C6—C11—N1	−0.5 (5)
C3—C2—C5—C6	176.7 (3)	C7—C6—C11—C10	0.3 (5)
O2—C5—C6—C11	−178.1 (3)	C5—C6—C11—C10	−178.8 (3)
C2—C5—C6—C11	4.8 (5)	C9—C10—C11—N1	−179.6 (3)
O2—C5—C6—C7	2.8 (5)	O3—C10—C11—N1	−0.3 (5)
C2—C5—C6—C7	−174.3 (3)	C9—C10—C11—C6	−1.3 (5)
C11—C6—C7—C8	0.6 (5)	O3—C10—C11—C6	178.0 (3)

### Hydrogen-bond geometry (Å, º)

**Table:**

*D*—H···*A*	*D*—H	H···*A*	*D*···*A*	*D*—H···*A*
N1—H1*N*···O1^i^	0.85 (2)	2.27 (3)	2.976 (4)	140 (3)
O2—H2···O1^ii^	0.82	1.94	2.665 (4)	147

Symmetry codes: (i) −*x*, −*y*+2, −*z*; (ii) *x*, *y*−1, *z*.
